# Extubation force depends upon angle of force application and fixation technique: a study of 7 methods

**DOI:** 10.1186/1471-2253-14-74

**Published:** 2014-08-24

**Authors:** Jennifer L Wagner, Robin Shandas, Craig J Lanning

**Affiliations:** 1 Department of Bioengineering, University of Colorado Denver, Aurora, CO, USA

**Keywords:** Unplanned extubation, Extubation force, Endotracheal tube fixation, Endotracheal tube restraint

## Abstract

**Background:**

Endotracheal tubes are frequently used to establish alternate airways. Precise placement of the tubes must be maintained to prevent serious complications. Several methods for fixation of endotracheal tubes are available. Available methods vary widely in form and functionality. Due to the unpredictable and dynamic nature of circumstances surrounding intubation, thorough evaluation of tube restraints may help reduce airway accidents such as tube dislodgement and unplanned extubation.

**Methods:**

Seven different tube-restraint combinations were compared against themselves and one another at a series of discrete angles (test points) covering a hemisphere on the plane of the face. Force values for tube motion of 2 cm and 5 cm (or failure) were recorded for 3 pull tests, at each angle, for each method of tube fixation.

**Results:**

All methods showed variation in the force required for tube motion with angle of force application. When forces were averaged over all test points, for each fixation technique, differences as large as 132 N (30 lbf) were observed (95% CI 113 N to 152 N). Compared to traditional methods of fixation, only 1 of the 3 commercially available devices consistently required a higher average force to displace the tube 2 cm and 5 cm. When ranges of force values for 5 cm displacement were compared, devices span from 80–290 N (18–65 lbf) while traditional methods span from 62–178 N (14–40 lbf), highlighting the value of examining forces at the different angles of application. Significant differences in standard deviations were also observed between the 7 techniques indicating that some methods may be more reproducible than others.

**Conclusions:**

Clinically, forces can be applied to endotracheal tubes from various directions. Efficacies of different fixation techniques are sensitive to the angle of force application. Standard deviations, which could be used as a measure of fixator reliability, also vary with angle of force application and method of tube restraint. Findings presented in this study may be used to advance clinical implementation of current methods as well as fixator device design in an effort to reduce the incidence of unplanned extubation.

## Background

Alternate airways are vital tools in emergency and critical care medicine as well as during procedures requiring anesthesia. Endotracheal (ET) tubes are regularly used to establish alternate airways. Proper placement and fixation of an ET tube is required to maintain efficacy of the airway. Several methods of ET tube restraint are available. Most sources recommend use of either adhesive tape, umbilical (non-adhesive) cotton twill tape or a commercially available tube holding device to secure an ET tube [1,2].

As little as 2 cm of ET tube motion can render an airway insufficient [3]. Due to the emergent and critical settings in which ET tubes are placed, a fast, reliable and easy to use method is highly valued [4]. Patient transport and routine care of critically ill patients also pose significant challenges to airway stability requiring frequent, focused efforts to maintain proper ET tube position. In addition to tube motion, unplanned extubation is common in critical care settings. Rates of unplanned extubation ranging from less than 1% to 43%, per intubated patient, have been reported [3,5-13]. Of these, one investigator estimated 92% of unplanned extubations were self-extubation with the remaining 8% being accidental extubations due to patient motion or unforeseen events such as coughing or equipment entanglement [13]. Another investigator produced similar results showing 15% self extubation and 85% accidental [14]. Unplanned extubation can lead to other complications such as: laryngeal injury, extended hospital stay, increased incidence of hospital acquired infections, epithelial tissue damage, vocal cord injury, bronchospasm, arrhythmias, respiratory arrest, insufficient oxygenation, anoxic brain injury and death [3,8,15,16]. More reliable, repeatable and easier to use methods of ET tube restraint would provide clinicians more confidence in the stability of alternate airways, as well as reduce complications arising from airway maintenance. Investigators have quantified several aspects of ET tube fixation over the last 50 years; however, questions still remain as to which type of fixation method is most effective. While previous studies have looked at extubation forces and compared methods of ET tube restraint, at the time of this work, no studies had looked at extubation force with respect to the angle of force application [4,17-22]. Clinically, extubation forces are applied from many different angles. Due to large variations in the form and functionality of various fixation methods, there is a need to examine several different angles of force application in order to thoroughly evaluate the performance of ET tube restraints. This investigation also provides an additional data set in a scarce body of research comparing and quantifying different methods of ET tube fixation.

It was hypothesized that forces required for ET tube displacement and extubation vary not only with method of fixation, but also with angle of force application. This study was designed to compare a given ET tube restraint to itself, over a series of angles, as well as compare different methods of ET tube restraint to one another. This study was designed to maintain consistency in test conditions and configuration across all methods of fixation and all angles by minimizing the number of variables controlled. Replication of clinical conditions such as body temperature and presence of saliva or perspiration would have introduced too many confounding factors for accurate comparisons across the methods and angles. By focusing on uniformity of fixator application and mannequin head position, this data set is suited to provide a starting point for ET tube restraint use and design optimization. Force values required for significant displacements, from different angles, can be combined with qualitative observations of failure modes to analyze performance of different tube fixation methods. This type of analysis can then be applied to the use of current techniques and devices as well as aid in the design of next generation ET tube restraints.

## Methods

**Figure 1:**
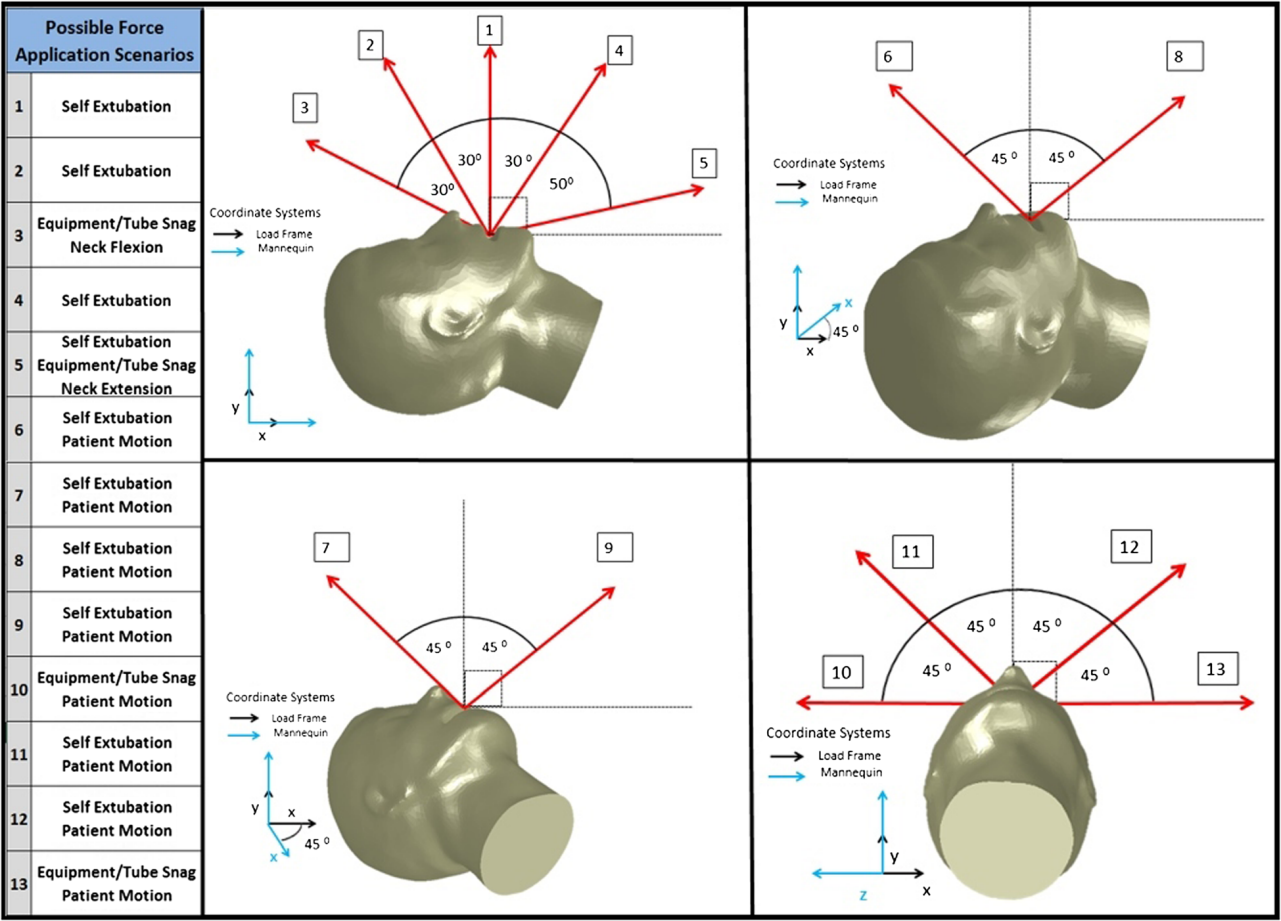
Depiction and description of the thirteen different angles tested along with potential clinical scenarios that could generate the given force vectors.

Forces required to displace an ET tube were measured at a series of discrete angles, encompassing nearly a full hemisphere, on the plane of the face. Angles were chosen to simulate possible extubation scenarios such as self-extubation, patient motion (neck flexion/extension, rolling), and equipment entanglement. All angles tested, along with possible clinical scenarios, are summarized in Figure 1. Three test pulls at each of the 13 angles (test points) were chosen for 2 reasons, material costs and statistical power. Initial testing performed by an independent lab on behalf of Securisyn Medical, LLC, revealed a sample size of 3 would provide enough data for comparisons to expose statistical significance where true differences in extubation force values were present.

**Figure 2:**
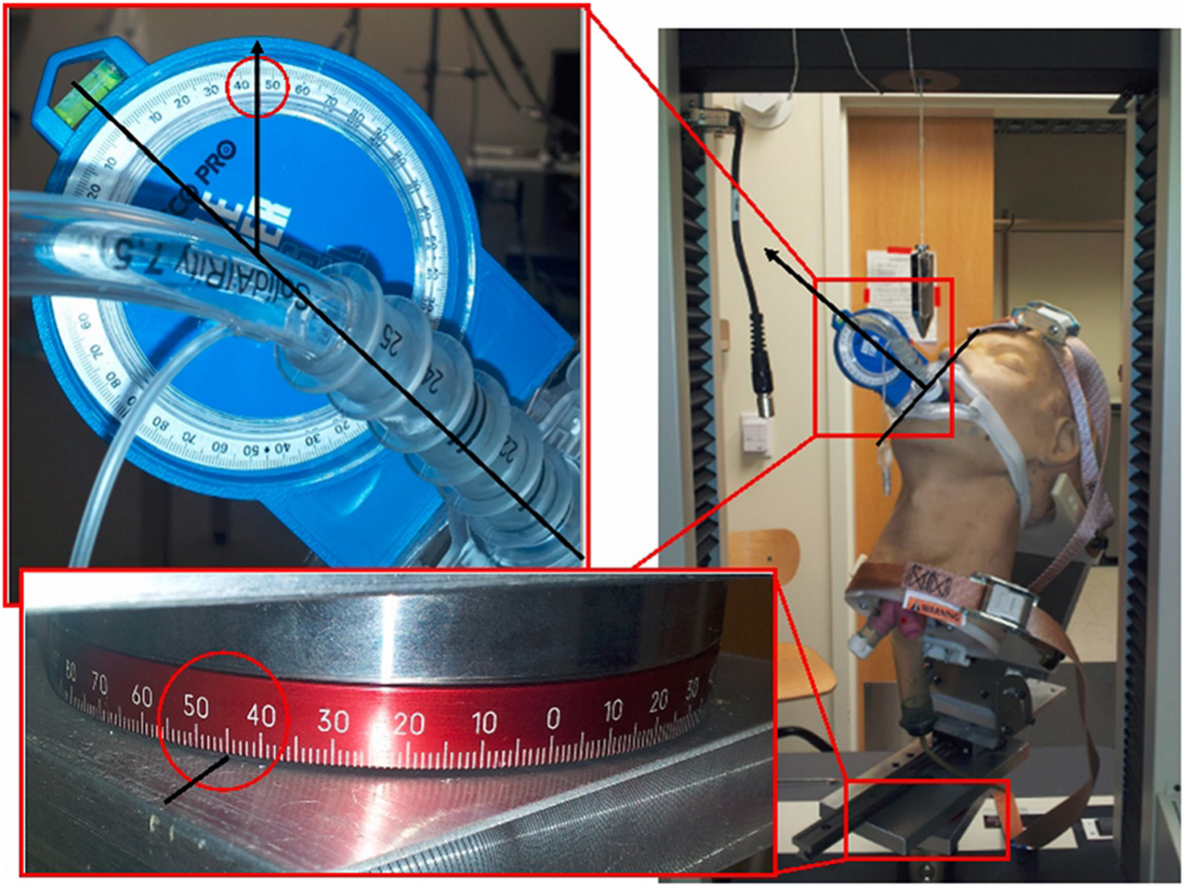
**Photograph of intubation mannequin in the test fixture/load frame assembly.** Setup corresponds to test point 7.

Angles at each of the 13 test points were achieved and maintained using a custom fixture designed to interface with a load frame and is shown in Figure 2. All testing was performed on a Laerdal intubation mannequin (Difficult Airway Trainer, Model 485-261-00101). Seven methods of ET tube restraint were tested using 2 different ET tubes. Currently, the most widely used method is adhesive tape [5,9,11,15,16]. For this study, the Lillehei method of applying adhesive tape was used to create a stabilization harness around the mannequin’s head. This method was tested on a traditional, cuffed ET tube (Mallinckrodt Hi-Lo Oral/Nasal Endotracheal Tube, cuffed 7.5 mm) as well as on a new, pre-market, Securisyn ET tube designed for use with a new fixation device, the SolidAIRity® system (Securisyn Medical, LLC). Another common stabilization technique uses umbilical (non-adhesive) cotton twill tape tied into a clove hitch knot around the ET tube. Both aforementioned methods of fixation were tested using both types of ET tubes. Significant differences between the Mallinckrodt and Securisyn tubes, such as radius of curvature and surface features, prompted testing both types of tubes with traditional tape and twill methods as observed differences could provide insight into the effects of tube geometry on ET tube restraint performance.

**Figure 3:**
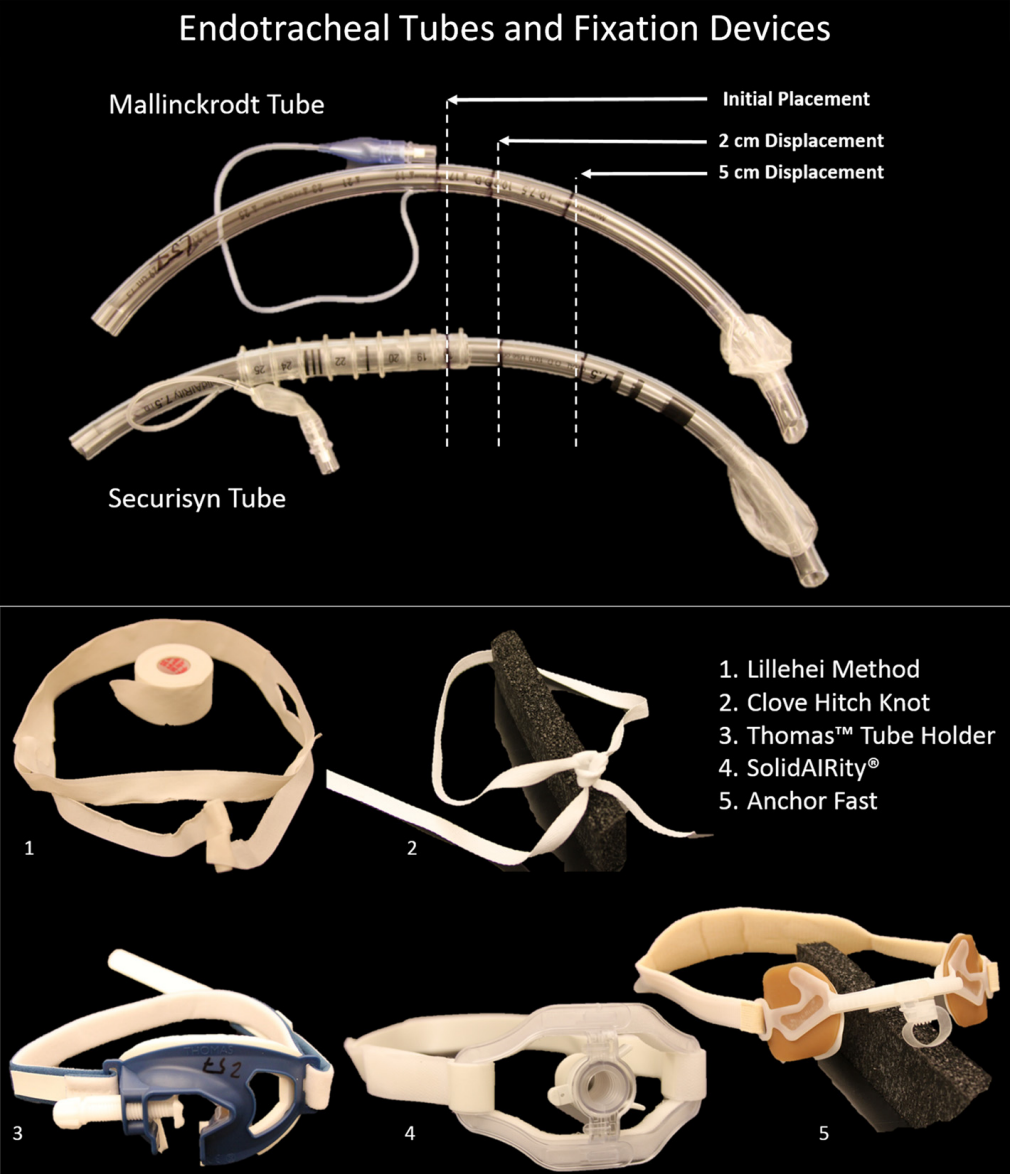
Endotracheal tubes and restraints used to create 7 different fixation combinations.

Common methods of ET tube fixation, such as adhesive tape or twill, can be cumbersome and time consuming to apply. Also, adhesive tape can lead to skin irritation and damage. Bodily fluids are present in critical care and emergent situation leading to difficulty when trying to use the more common, traditional methods of tube fixation. Commercially available fixation devices aim to reduce time associated with ET tube restraint application and maintenance while increasing repeatability and reliability of fixator installation and performance. Thomas™ tube holders and Anchor Fast (Hollister) devices were both tested using Mallinckrodt ET tubes while the SolidAIRity® system was tested using Securisyn tubes. All devices were placed on the mannequin according to manufacturer’s instructions.Forces were measured using a 2 kN load cell (MTS Model #569327-08) in an MTS 5 kN load frame and rubber-faced clamp grip (MTS Part # 121–001). Once secured in the grip, ET tubes were displaced vertically at a rate of 7.62 cm/min (3 in/min). Force values were manually recorded at 2 cm and 5 cm displacement or device failure. Displacement was determined by observing markings made on the ET tube relative to the mannequin’s lower incisors. Markings were made on the tube by first marking the initial placement level (18 cm) as measured along the length of the tube from the distal end. Next, marks were made at 16 cm and 13 cm, representing 2 cm and 5 cm displacement respectively. Both tubes and all fixation methods are pictured in Figure 3.

Stabilization devices and tape lengths were controlled by measuring and marking tensioning devices and tape lengths. All ET tubes and stabilizers were marked and placed by a single investigator using a laryngoscope (Welch Allyn 69504). All measurements were observed and recorded by a single operator. After placement, all ET tube cuffs were inflated with 15 cc of air using a 20 cc syringe. New tubes and devices were used for every pull test. A total of 3 pulls were performed, using each stabilization technique, at each angle. To ensure positioning consistency, the test fixture was set into position for a given test point then remained undisturbed until all pulls, for all methods, were completed.

## Results

**Figure 4:**
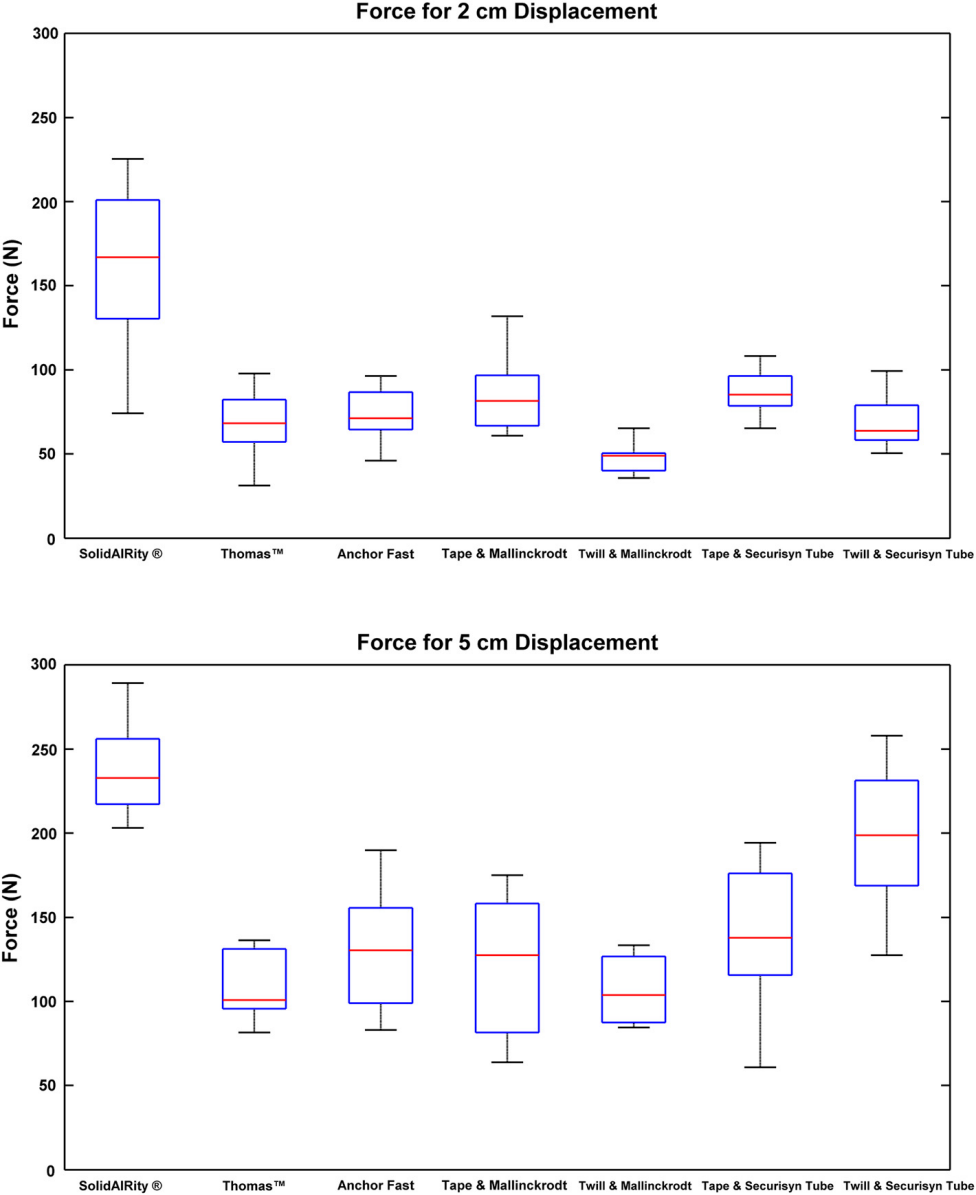
**Boxplot of force data grouped by method of fixation.** Red line indicates median value, box indicates interquartile range and bars show min/max values.

Variations in force values were observed across angles, for a given device, as well as across the 7 different methods of fixation. Standard deviations between test pulls were also observed to depend upon the angle of force application and fixation method. In general, both force values and standard deviations exhibited a strong dependence upon angle of force application. Asymmetries in boxplots of the data (Figure 4) indicate uneven distribution and a skewed dataset. Qualitative analysis of the dataset, using both boxplots and scatter plots (see Additional file 1: Figure S1) indicated a high likelihood of unequal variances. Given this fact, coupled with a small sample size, statistical analysis was limited to t-tests which were performed assuming unequal variance.

Forces were compared at each of the 13 test points. Three test values were used to determine a mean force and standard deviation at each test point. Means and standard deviations for each fixation technique were then calculated for all 39 force values (3 pulls at 13 angles) to allow for comparison of fixation methods across all test points. Differences between fixation methods were analyzed using a 2 tailed t test (α = 0.05). Both mean force values and standard deviations were analyzed using MATLAB’s "ttest2" function (MATLAB version R2013a). To account for the unequal and uneven distributions described above, the option for unequal variance was used.

**Figure 5:**
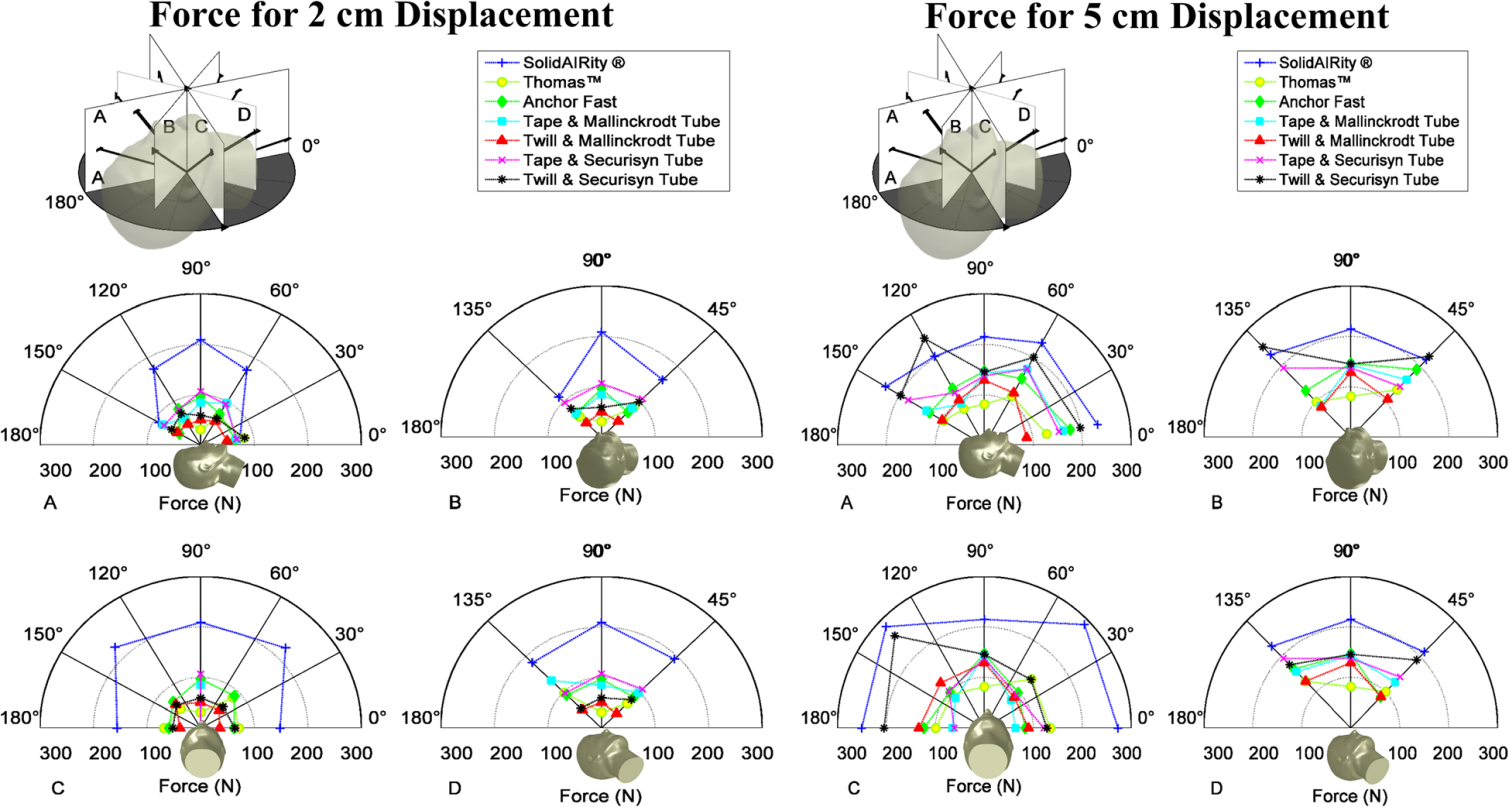
**Illustration of average of 3 force measurements with respect to mannequin head.** Average of all 3 force measurements, at each angle, for 2 cm and 5 cm tube displacement.

Confidence intervals (95%, α = 0.05) were also calculated for the differences between mean force values and mean standard deviations. Confidence intervals were also calculated using the "ttest2" function with the unequal variance flag set. This calculation makes use of the Satterthwaite’s approximation for degrees of freedom, sometimes referred to as Welch’s *t*-test. Figure 5 provides a graphical illustration of the dataset as it relates to the angle of force application. Force values displayed were generated by averaging the three test pulls at each test point for both 2 cm and 5 cm displacement. Discrepancies between force values from one test point to another provide insight into the fixator mechanisms strengths and weaknesses. For example, cotton twill tends to have higher force values at angles 45° from center while some of the devices required the most force when the force was applied directly from center (test point 1).

Table 1 presents mean force values and standard deviations for all methods of fixation at each test point, as well as an average of test point means for each method. Generally speaking, traditional methods of securing the tube required similar forces for displacing the tube as the commercially available devices, with exception of the SolidAIRity® system. For clinically significant displacement (2 cm) adhesive and twill tape methods required approximately 82 N while the SolidAIRity®, Thomas™ and Anchor Fast systems required 165 N, 67 N and 74 N respectively. Force values at each angle provide insight into strengths and weakness of each method’s unique mechanics, further addressed in the discussion section.

**Table 1:** Average forces required to displace tubes both 2 cm and 5 cm or fixation failure

	**Average force for 2 cm displacement (N)**													
	**SolidAIRity®**		**Thomas™**		**Anchor Fast**		**Tape & Mallinckrodt**		**Twill & Mallinckrodt**		**Tape & Securisyn tube**		**Twill & Securisyn tube**	
**Test point**	**Mean**	**Standard deviation**	**Mean**	**Standard deviation**	**Mean**	**Standard deviation**	**Mean**	**Standard deviation**	**Mean**	**Standard deviation**	**Mean**	**Standard deviation**	**Mean**	**Standard deviation**
**1**	209	0.00	31	7.70	96	6.79	85	26.69	50	13.59	107	23.11	50	6.79
**2**	175	12.84	58	26.69	83	2.57	61	20.55	47	2.57	82	11.19	73	2.57
**3**	90	6.79	55	5.14	46	2.57	82	9.26	50	10.27	79	28.25	62	8.90
**4**	172	13.59	65	5.14	71	4.45	96	11.19	55	15.62	93	4.45	61	5.14
**5**	74	18.52	82	2.57	65	2.57	67	0.00	50	6.79	68	2.57	83	2.57
**6**	193	2.57	68	11.19	86	6.79	98	19.39	40	0.00	108	28.94	79	2.57
**7**	162	13.59	85	7.70	70	6.79	82	20.06	44	0.00	79	13.59	99	13.59
**8**	200	30.82	98	7.70	92	5.14	132	5.14	50	5.14	96	16.84	55	6.79
**9**	113	22.83	58	7.70	67	4.45	67	27.06	40	7.70	89	20.38	80	4.45
**10**	156	4.45	68	2.57	59	2.57	65*	13.59	39	2.57	61*	18.52	52	6.79
**11**	225	9.26	53	4.45	73	2.57	83*	29.62	65	2.57	102*	31.14	64	6.79
**12**	224	2.57	85	20.38	89	4.45	77*	14.30	49	23.11	92*	21.02	59	5.14
**13**	148	5.14	71	4.45	62	8.90	64*	11.19	36	0.00	65	9.26	64	5.14
**Average**	**165**	**11.00**	**67**	**8.72**	**74**	**4.66**	**81**	**16.00**	**47**	**6.92**	**84**	**16.73**	**68**	**5.94**
	**Average force for 5 cm displacement or failure (N)**													
**1**	215	6.79	82	12.84	147	15.41	144	21.02	129	24.77	138	27.06	145	40.36
**2**	203	26.81	83	18.52	130	6.79	95	2.57	104	31.56	120	33.58	245	11.77
**3**	233	16.84	96	11.19	129	0.00	136	5.14	99	12.84	179	16.84	197	28.94
**4**	234	13.59	113	11.19	153	24.50	175	24.50	120	22.24	175	6.79	202	21.94
**5**	234	20.06	129	8.90	178	35.31	163	21.02	87	12.84	154	33.97	199	35.95
**6**	212	13.59	101	9.26	86	43.66	128	5.14	87	14.30	142	42.90	190	24.50
**7**	218	11.77	133	4.45	190	25.68	162	6.79	107	19.39	193	31.56	227	29.17
**8**	228	21.02	130	5.14	165	29.17	157	2.57	130	28.94	194	32.18	176	56.50
**9**	231	8.90	99	9.26	130	6.79	87	22.39	85	30.82	128	26.06	254	32.08
**10**	251	15.62	99	26.06	123	11.19	65	13.59	133	4.45	61	18.52	205	4.45
**11**	283	12.84	93	16.04	99	30.28	83	29.62	126	10.27	102	31.14	258	31.14
**12**	289	4.45	136	20.06	98	11.77	77	14.30	86	9.26	92	21.02	135	13.59
**13**	273	17.98	135	25.29	83	5.14	64	11.19	90	12.84	122	17.98	128	20.06
**Average**	**239**	**14.64**	**110**	**13.71**	**132**	**18.90**	**118**	**13.83**	**107**	**18.04**	**138**	**26.12**	**197**	**26.96**

Mean and standard deviation for all 3 measured force values, at all 13 test points, for 7 methods of ET tube restraint. Values listed in the last row of the table are the average values for all test point means and standard deviations.

*Indicates fixator failed before 2 cm displacement was reached.

It is interesting to note the Lillehei method of taping the tube failed before 2 cm displacement at test points 10–13, using the Mallinckrodt tube, and test points 10–12 using the Securisyn tube. This means that the tape tore before the tube underwent significant displacement. From this data, it can be reasoned that adhesive tape is particularly effective at limiting tube motion against forces in the lateral direction, given force are below a certain threshold of around 60–100 N (see Table 1).

In the case of extubation (5 cm displacement) the same trend of traditional methods comparing to devices was observed with one exception. The Securisyn tube and twill tape combination required significantly more force for displacement than the twill and Mallinckrodt combination (197 N vs 107 N). This is due to the way the knot would position on the ribs allowing the knot to maintain tension for the duration of the pull. When comparing standard deviations, commercially available devices consistently exhibited lower values for both 2 cm and 5 cm displacement. Standard deviations for traditional methods ranged from 6–27 N while the device standard deviations ranged from 5–19 N.

The SolidAIRity® system consistently required the highest force values for significant displacement. Force values as much as 3 times greater than other methods were observed at some test points. When comparing SolidAIRity® to other methods at both 2 cm and 5 cm displacement force values were significantly higher than all other methods. Statistical results comparing differences between mean force values and standard deviations across all test points, for a given device, are summarized in Tables 2 and 3. Mean force values in these tables were calculated as the mean of all 39 force values for each device. Some significant differences were also observed between standard deviations of the different methods of fixation. The Anchor Fast system exhibited the smallest standard deviations with 3 of the 6 comparisons against other methods demonstrating statistical significance (p < 0.05). When compared to 2 cm displacement, 5 cm displacement values had less significant, but larger magnitude, differences when comparing the standards deviations of the different methods.

**Table 2:** Statistical comparison of force values for 2 cm tube displacement

**Comparison of force values at 2 cm displacement (N)**								
	**SolidAIRity®**	**Thomas™**	**Anchor Fast**	**Tape & Malllinckrodt**	**Twill & Mallinckrodt**	**Tape & Securisyn**	**Twill & Securisyn**	
**SolidAIRity®**		**0.000**	**0.000**	**0.000**	**0.000**	**0.000**	**0.000**	**P values**
**Thomas™**	97 [61.99; 126.68]		0.320	0.057	**0.001**	**0.009**	0.957	
**Anchor Fast**	91 [55.90; 120.00]	-6 [-19.37; 6.60]		0.188	**0.000**	**0.052**	0.296	
**Tape & Malllinckrodt**	83 [42.52; 110.28]	-14 [-36.48; 0.62]	-8 [-29.49; 6.40]		**0.001**	0.887	**0.053**	
**Twill & Mallinckrodt**	117 [82.75; 145.84]	20 [8.83; 31.10]	26 [16.70; 36.02]	34 [20.90; 54.88]		**0.000**	**0.000**	
**Tape & Securisyn**	78 [42.84; 107.46]	-19 [-33.13; -5.24]	-12 [-25.74; 0.15]	-5 [-19.76; 17.25]	-39 [-50.31; -27.98]		**0.006**	
**Twill & Securisyn**	97 [61.99; 126.00]	0 [-13.18; 12.50]	6 [-5.63; 17.72]	14 [-0.27; 35.45]	-20 [-29.75; -10.86]	18 [6.04; 31.64]		
	**Difference between means and 95% confidence interval**							
**Difference in standard deviation of means at 2 cm displacement**								
	**SolidAIRity®**	**Thomas™**	**Anchor Fast**	**Tape & Malllinckrodt**	**Twill & Mallinckrodt**	**Tape & Securisyn**	**Twill & Securisyn**	
**SolidAIRity®**		0.481	**0.027**	0.166	0.211	0.124	0.074	**P values**
**Thomas™**	2.27 [-4.30; 8.85]		0.068	**0.030**	0.522	**0.022**	0.212	
**Anchor Fast**	6.33 [0.822; 11.85]	4.06 [-0.35; 8.47]		**0.001**	0.288	**0.001**	0.223	
**Tape & Malllinckrodt**	-5.00 [-12.23; 2.22]	-7.28 [-13.81; -0.75]	-11.3 [-16.79; -5.89]		**0.008**	0.840	**0.002**	
**Twill & Mallinckrodt**	4.08 [-2.48; 10.64]	1.8 [-3.93; 7.54]	-2.26 [-6.64; 2;12]	9.08 [2.56; 15.60]		**0.006**	0.652	
**Tape & Securisyn**	-6.64 [-13.16; 1.69]	-8.91 [-14.76; -1.26]	-12.97 [-17.81; -6.33]	-1.63 [-8.11; 6.65]	-10.72 [-16.55; -3.08]		**0.001**	
**Twill & Securisyn**	5.06 [-0.55; 10.66]	2.8 [-1.75; 7.32]	-1.28 [-3.40; 0.84]	10.06 [4.51; 15.61]	0.98 [-3.53; 5.48]	11.7 [4.96; 16.62]		
	**Difference between means and 95% confidence interval**							

Statistical analysis of force values averaged across all test points for each fixation technique at 2 cm measured displacement. Confidence intervals were calculated using the Satterthwaite’s approximation (Welch’s *t*-test) due to likelihood of unequal variance. Difference values are the result of subtracting the mean value for the method in the row from the mean value for the method in the column (column mean-row mean). Differences showing statistical significance (p < 0.05) are listed in bold.

**Table 3:** Statistical comparison of force values for 5 cm tube displacement or failure of fixator

**Comparison of force values at 5 cm displacement or failure (N)**								
	**SolidAIRity®**	**Thomas™**	**Anchor Fast**	**Tape & Malllinckrodt**	**Twill & Mallinckrodt**	**Tape & Securisyn**	**Twill & Securisyn**	
**SolidAIRity®**		**0.000**	**0.000**	**0.000**	**0.000**	**0.000**	**0.007**	**P values**
**Thomas™**	129 [109.13; 148.41]		0.065	0.530	0.651	0.036	**0.000**	
**Anchor Fast**	107 [82.07; 132.35]	-22 [-44.59; 1.47]		0.371	**0.032**	0.645	**0.000**	
**Tape & Malllinckrodt**	121 [92.35; 149.00]	-8 [-34.66; 18.46]	13 [-17.01; 43.93]		0.365	0.214	**0.000**	
**Twill & Mallinckrodt**	132 [113.09; 151.53]	4 [-12.39; 19.46]	25 [2.40; 47.49]	12 [-14.66; 37.92]		**0.019**	**0.000**	
**Tape & Securisyn**	100 [72.22; 128.52]	-28 [-54.78; -2.02]	-7 [-37.17; 23.48]	-20 [-53.13; 12.53]	-32 [-58.04; -5.83]		**0.002**	
**Twill & Securisyn**	42 [12.60; 71.35]	-87 [-114.51; -59.08]	-65 [-96.67; -33.81]	-79 [-112.52; -44.87]	-90 [-117.79; -62.88]	-58 [-92.10; -24.70]		
	**Difference between means and 95% confidence interval**							
**Difference in standard deviation of means at 5 cm displacement or failure**								
	**SolidAIRity ®**	**Thomas™**	**Anchor Fast**	**Tape & Malllinckrodt**	**Twill & Mallinckrodt**	**Tape & Securisyn**	**Twill & Securisyn**	
**SolidAIRity ®**		0.723	0.311	0.795	0.267	**0.002**	**0.008**	**P values**
**Thomas™**	0.93 [-4.40; 6.26]		0.232	0.969	0.181	**0.001**	**0.006**	
**Anchor Fast**	-4.26 [-12.90; 4.36]	-5.19 [-14.00; 3.62]		0.273	0.849	0.128	0.140	
**Tape & Malllinckrodt**	0.80 [-5.54; 7.14]	-0.13 [-6.74; 6.49]	5.07 [-4.29; 14.42]		0.246	**0.003**	**0.008**	
**Twill & Mallinckrodt**	-3.40 [-9.62; 2.81]	-4.33 [-10.83; 2.17]	0.86 [-8.42; 10.14]	-4.21 [-11.5; 3.09]		**0.035**	0.060	
**Tape & Securisyn**	-11.49 [-18.05; -4.92]	-12.42 [-19.25; -5.59]	-7.22 [-16.71; 2.26]	-12.29 [-19.87; -4.71]	-8.08 [-15.56; -0.60]		0.857	
**Twill & Securisyn**	-12.32 [-21.00; -3.65]	-13.5 [-22.10; -4.40]	-8.06 [-18.94; 2.82]	-13.12 [-22.51; -3.73]	-8.92 [-18.24; 0.40]	-0.83 [-10.35; 8.69]		
	**Difference between means and 95% confidence interval**							

Statistical analysis of force values averaged across all test points for each fixation technique at 5 cm measured displacement. Confidence intervals were calculated using the Satterthwaite’s approximation (Welch’s *t*-test) due to likelihood of unequal variance. Difference values are the result of subtracting the mean value for the method in the row from the mean value for the method in the column (column mean-row mean). Differences showing statistical significance (α < 0.05) are listed in bold.

### Failure modes

Each method of fixation had a unique failure mode. For the purpose of this discussion, failure is defined as either failure of fixator integrity or failure of the fixation technique to prevent 5 cm of ET tube displacement. The SolidAIRity® system failed by either the tube ribs slipping through the stabilizer ribs or due to fracture of the stabilization device. Thomas™ tube holders would allow tubes to slip to the side of the clamp while Anchor Fast devices consistently failed at the attachment point of the sliding member to the bar, either by fracture of the clips or sliding off of the bar. Adhesive tape tore either between the cheek and tube or at the tube-tape interface while the knot used for twill tape fixation tended to loose tension.

Failure analysis serves to aid in evaluating the underlying mechanics of each method. Understanding failure modes may assist in determining which method of fixation may be most effective in a particular patient care setting. A summary of the failure modes is provided in Table 4.

**Table 4:** Observed failure modes for all methods of fixation

**Mode**	**SolidAIRity®**	**Thomas™**	**Anchor Fast**	**Tape & Mallinckrodt tube**	**Twill & Mallinckrodt tube**	**Tape & Securisyn tube**	**Twill & Securisyn tube**
**1**	**Tube slipped through stabilizer:** Ribs on the tube would deform and flatten out enough to slide through the stabilization device. Loading from any angles except center would sometimes cause the stabilizer parts to separate (splay) enough for the tube to slip through (Test points 1, 2, 3, 4, 6, 9, 11, 12).	**Tube slipped through clamp:** This mode of failure did not result in any damage to the device rather it just failed to prevent tube motion by allowing the tube to slide through the clamp mechanism.	**Clip broke at attachment point of sliding member:** A feature for lateral adjustment of tube placement is included with this device. It consists of a clip that slides along a bar. This clip would fail catastrophically, with pieces breaking off (test points 1, 2, 3, 4, 6, 7, 8, 9, 11).	**Tape tore between tube and cheek:** All angles demonstrated same failure mechanism of tape tearing.	**Knot lost Tension:** All failures were the same.	**Tape tore between tube and cheek or at tape-tube interface:** All angles demonstrated same failure mechanism of the tape tearing where it came in contact with the ribs of the ET tube.	**Knot lost Tension:** All tests that failed exhibited a gradual loss in knot tension and progressive slipping of the tube through the knot. Certain angles allowed the knot to maintain tension generating very large force values (test points 2, 7, 9, 11).
**2**	Depending upon angle of force application, at very large loads (>200 N) sometimes the entire stabilization device would fail by cracking of the plastic stabilization pieces (Test points 5, 7, 8, 10, 13).		**Clip slid off bar:** Alternatively, the clip mechanism would fail by sliding off the end of the bar on which it travels (test point 13) or both slide off and break (test point 10).				

Description of failure modes for all methods of ET tube fixation.

It should also be noted that the devices (as opposed to tradition tape and twill methods) tended to provoke occlusion events prior to extubation at the more extreme test angles (test points 10 and 13). For example, when forces were applied laterally, rigid forms of stabilization (devices) caused the tube to bend and occlude while traditional methods were observed to undergo larger lateral deviaitons. Due to the nature of fixation being more of a point on the tube rather than immobilization of a segment of the tube, traditional methods allow for rotation while, at extreme angles of force application, devices do not.

## Discussion

Traditional methods of ET tube fixation required similar extubation forces as commercially available devices with one exception. Generally speaking, the SolidAIRity® system requires the largest force to displace the tube. Interestingly, the highest recorded force value (240 N) was measured at test point 11 using the Securisyn tube and twill tape. This is likely due to the clove hitch knot being in an ideal position, with respect to the force vector, to maintain tension. Conversely, standard deviation’s were higher for traditional methods when compared to devices, perhaps speaking to the ease of device use, application and repeatability with respect to things like bodily fluids and patient position.

Evaluating a fixation method across a series of angles provides a quantitative framework with which fixation techniques may be optimized and weaknesses identified. Standard deviation could be used as a measure of fixator reliability. For example, some methods, such as adhesive tape, twill tape and the Thomas™ tube holder, are difficult to secure the same way every time. It is easier to ensure consistency in a laboratory setting, so one may expect standard deviations to be higher in a clinical setting. Devices offer faster application, with less steps, leading to more reproducible application and arguably more reliability.

Also, a high force value for displacement is not the sole indication of an effective method. For example, the highest force value recorded using twill tape and a Securisyn tube prevented displacement, but the knot had so much tension that the tube was occluded, preventing air flow. Body mechanics also play a role in the force values. For example, motion of the mandible in response to a certain device may induce large displacements relative to the incisors, but not to the vocal cords. Motion of the mandible can absorb rotations and displacements, as well as shift locations of fulcrums, resulting in variations of fixator performance not only across angle of force application, but when compared to other methods in similar loading conditions.

Comparison between methods offers insight into the mechanics of ET tube fixation. Using adhesive tape and the Lillehei method, the tube is allowed to rotate in all axes about the point of tape application while the SolidAIRity® system secures the tube along a length of approximately 3.81 cm (1.5 in), allowing tube rotation only about the long axis of the tube. This fundamental difference in design translates into differences in force, and moment, distributions and ultimately measured force values for a given displacement. Specifically, at test points 3 and 5, 2 cm of motion was observed at a relatively lower force value for the SolidAIRity® system due to the fact that the entire device had to rotate about the mouth. After initial displacements and rotations it took much more force, relative to other test points, to displace the tube 5 cm. Conversely, at test points 3 and 5, using the Lillehei method with adhesive tape required an unremarkable force to reach 2 cm displacement but a relatively low force for 5 cm displacement due to the fact that the tape was seeing stresses at approximately 45°, producing maximum shear stress values resulting in tape failure.

Asymmetries and interfaces in fixation techniques create opportunities for systemic weakness as well. When forces were applied to the Anchor Fast system from the lateral directions, such as test points 6, 10 and 13, the tube interface would tend to slide off of the bar member designed to allow for lateral tube motion. At other test points, such as 1, 2 and 3, the clips attaching the tube interface to the slide bar would quickly snap off in response to a 45° force angle on the clip. When testing the Thomas™ tube holder at test point 10, the tube would slip to the side of the clamp allowing for low force extubation. These examples of asymmetric failure illustrate the value of testing across multiple force vectors.

Three similar studies have been published since 2009. Values measured in this study agree reasonably well with the previous studies conducted by Carlson [18] in 2007, Owen [19] in 2009, and Shimizu [17] in 2011. Carlson reported force values ranging from 67–111 N for 1" adhesive tape and an average force of 165 N for the Thomas™ tube holder. Testing was conducted on cadavers with forces applied perpendicular to the face and extubation defined as when the tube cuff advanced beyond the vocal cords. Owen et al. reported force values of 58 ± 18 N for the Thomas™ tube holder, 33 ± 23 N for a non-adhesive tape and 87 ± 34 N for 1" adhesive tape. For said study, forces were applied perpendicular to the face with extubation defined as 7 cm of tube displacement. Shimizu et al. reported 106 ± 4 N for the Thomas™ tube holder and 131 ± 12 N for 1" adhesive tape. Extubation was defined as when the tube cuff advanced beyond the vocal cords with forces applied perpendicular to the face.

Testing done on a mannequin at room temperature omits certain effects present in a living patient such as temperature gradients, gender, facial hair, perspiration and saliva. Material properties, and subsequent force responses, may change significantly when exposed to a temperature gradient. This could translate into increased ET tube or fixator deformation and/or weakening of tube-restraint interfaces, as well as interfaces internal to the fixator. Significant differences between genders are also present in the facial area. Factors such as bone and skin structure or the presence/absence of facial hair are not accurately represented by an intubation mannequin. On the other hand, the mannequin is useful for controling the external factors mentioned above leaving the study less prone to confounding factors. Perspiration is certainly present on some patients, particularly those in the ICU or ED. An absence of saliva or perspiration may also effect fixator performance. These effects would likely not be consistent across devices, or even angles of force application, so relativistic observations made in this study may not necessarily hold true in the clinical setting.

Clinically, the nature of the force will also vary widely from impulse forces to long term, constant pressures. Materials and fixation mechanisms could behave differently under different loading conditions. Material properties could also change significantly after exposure to long term loading, deformation and temperature gradients. Application of ET tube restraints may also vary significantly between patients, particularly for the adhesive and twill tapes, as the fixators are essentially constructed on, versus applied to, the patient.

It is likely that there is also human error present in the displacement readings. Motion of the mandible, with respect to the tube and mannequin, was not consistent across all methods or test points. It is possible errors were induced determining alignment of the ET tube markings with the incisors. All markings were made by hand, so there may also be some error inherent in the tube markings.

## Conclusions

Force data could be used to optimize use, and development of new, methods for endotracheal tube fixation. Efficacy of various methods of endotracheal tube restraint are dependent upon the angle at which the extubation force is applied. This information, coupled with an analysis of standard deviations for several methods across several angles, provides insight into reliability of one method versus another. Comparison of test angles may also be compared with clinical scenarios to help guide usage of one method versus another for a given airway management setting such as transport, long term critical care or intra-operative.

Further analysis of data gathered during this study could be conducted to aid in optimization of endotracheal tube fixator design and use. This data could also be used to evaluate current practices with respect to equipment management and logistics, ET tube restraint implementation and use of different fixation techniques in different situations.

In the future, it may be valuable to explore the effects of environment on tube properties and fixator mechanics. Inclusion of variables such as temperature, patient features and bodily fluids would be a logical next step in investigation fixator performance. Another valuable addition would be variations in applied force. Repeating this study with an impulse force may reveal weakness in fixator performance not visible in this study.

### Key messages

• Force data can aid in optimization of use, and development of new, methods for endotracheal tube fixation

• Forces required to displace an endotracheal tube depend upon the angle at which the forces are applied

• Force values and standard deviations for extubation vary significantly with method used and angle of applied force

• When evaluating methods of endotracheal tube restraint, standard deviation could be used as a measure of method reliability

• Significant differences in standard deviations were observed between methods of fixation and angle of force application

## Competing interests

Funding for materials (endotracheal tubes, restraint devices, test fixation device construction) and personnel time (design and fabrication of test and required equipment, data collection) were provided, in part, by Securisyn Medical, LLC. Time for data analysis and manuscript generation was paid for solely by the University of Colorado, Denver Department of Bioengineering. All data collection, analysis and manuscript generation were performed solely by the authors of this manuscript.

## Authors’ contributions

RS secured funding and provided guidance and management throughout all phases of this study. CL provided equipment and software training. JW, RS and CL participated in study conception, design, execution, analysis and manuscript organization. JW designed the test fixture and performed all data collection. JW, CL and RS participated in data analysis. Manuscript was prepared by JW. JW, RS and CL all read and approved this manuscript for submission.

## Pre-publication history

The pre-publication history for this paper can be accessed here:

http://www.biomedcentral.com/1471-2253/14/74/prepub

## Supplementary Material

Additional file 1: Figure S1 Scatter plot of entire dataset. Force values for tests that failed before 2 cm displacement appear only in the 5 cm or series. Click here for file
