# Supplementary figures and images for: Extubation force depends upon angle of force application and fixation technique: a study of 7 methods
Source: BMC Anesthesiol. 2014 Aug 24;14:74. doi: 10.1186/1471-2253-14-74 (PMC4161264; doi:10.1186/1471-2253-14-74)

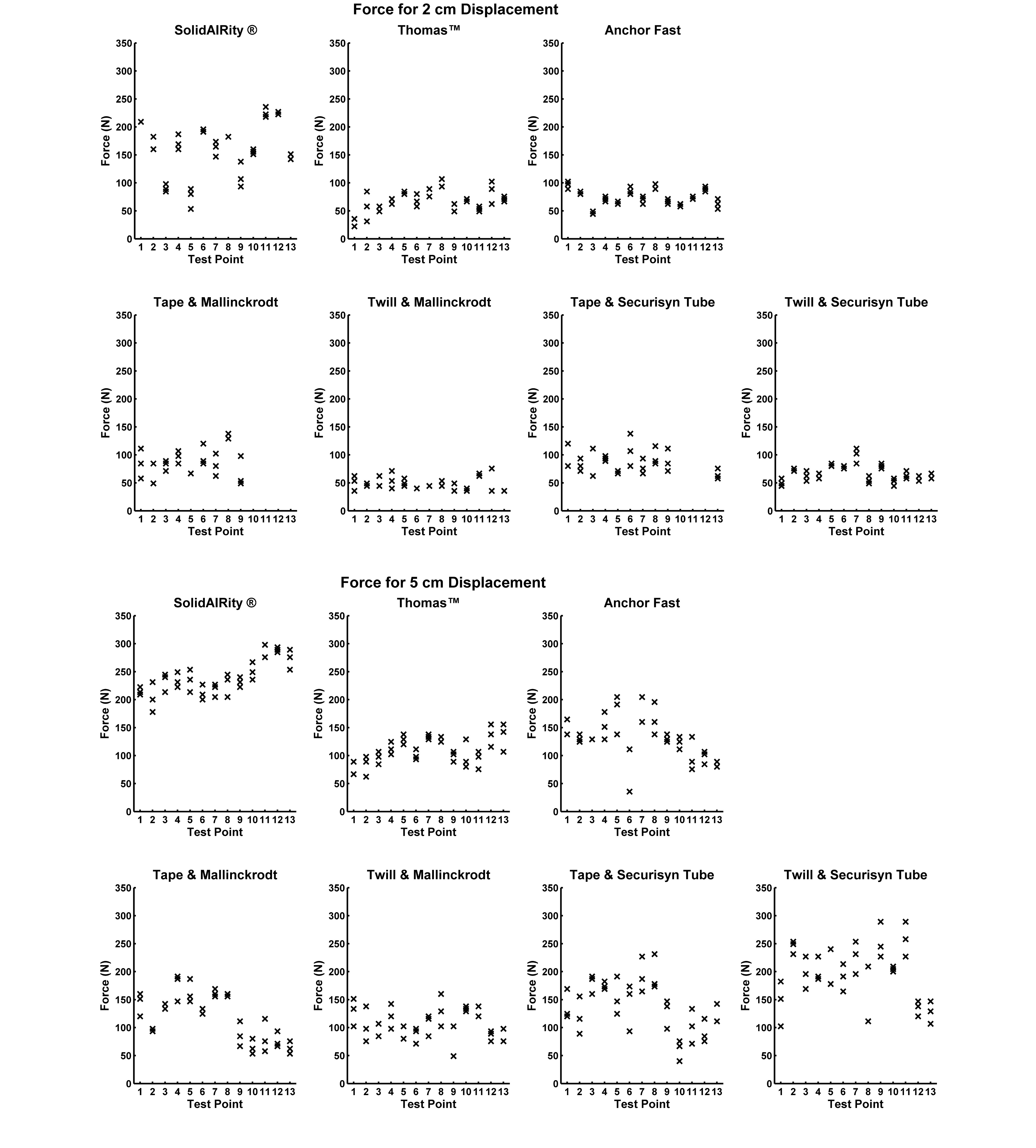

Supplement: Additional file 1: Figure S1 — Scatter plot of entire dataset. Force values for tests that failed before 2 cm displacement appear only in the 5 cm or series. [file 1471-2253-14-74-S1.tiff]
